# Effect of Root Restriction on the Growth, Photosynthesis Rate, and Source and Sink Relationship of Chilli (*Capsicum annuum* L.) Grown in Soilless Culture

**DOI:** 10.1155/2020/2706937

**Published:** 2020-01-28

**Authors:** Nurul Idayu Zakaria, Mohd Razi Ismail, Yahya Awang, Puteri Edaroyati Megat Wahab, Zulkarami Berahim

**Affiliations:** ^1^Laboratory of Climate-Smart Food Crop Production, Institute of Tropical Agriculture and Food Security, Universiti Putra Malaysia, 43400 Serdang, Selangor, Malaysia; ^2^Department of Crop Science, Faculty of Agriculture, Universiti Putra Malaysia, 43400 Serdang, Selangor, Malaysia

## Abstract

Chilli (*Capsicum annum* L.) plant is a high economic value vegetable in Malaysia, cultivated in soilless culture containers. In soilless culture, the adoption of small container sizes to optimize the volume of the growing substrate could potentially reduce the production cost, but will lead to a reduction of plant growth and yield. By understanding the physiological mechanism of the growth reduction, several potential measures could be adopted to improve yield under restricted root conditions. The mechanism of growth reduction of plants subjected to root restriction remains unclear. This study was conducted to determine the physiological mechanism of growth reduction of root-restricted chilli plants grown in polyvinyl-chloride (PVC) column of two different volumes, 2392 cm^3^(root-restricted) and 9570 cm^3^(control) in soilless culture. Root restriction affected plant growth, physiological process, and yield of chilli plants. Root restriction reduced the photosynthesis rate and photochemical activity of PSII, and increased relative chlorophyll content. Limited root growth in root restriction caused an accumulation of high levels of sucrose in the stem and suggested a transition of the stem as a major sink organ for photoassimilate. Growth reduction in root restriction was not related to limited carbohydrate production, but due to the low sink demand from the roots. Reduction of the total yield per plant about, 23% in root restriction was concomitant, with a slightly increased harvest index which reflected an increased photoassimilate partitioning to the fruit production and suggested more efficient fruits production in the given small plant size of root restriction.

## 1. Introduction

Chilli (*Capsicum annuum* L.) is one of the major high value vegetable crops cultivated in Malaysia, mainly for its pungency. Chilli is rich in vitamin E, vitamin C, and *β*-carotene [1]. Currently, the self-sufficiency level of chilli in Malaysia is only at 51.4%, which contributes significantly to the high food import cost [2]. Soilless culture under protected structure has been used sporadically in Malaysia for commercial vegetable production to improve plant growth, yield, and income [3]. Soilless culture technique depends largely on the use of polybags filled with a growing substrate, such as coconut coir dust [4]. Coconut coir dust may become a limited resource and more expensive in the future due to the high demand of consumption, while inefficient use of this substrate will lead to a higher production cost [5].

The substrate can be efficiently used in a small container size; however, from previous study, chilli plants grown in the small container had shown a reduction in shoots and root growth, which could not have been caused by water or nutrient stress [6]. Reduced plant growth in small container sizes may be caused by a diminished ability of the plant to accumulate photosynthates continually in the sink organ, including roots [7]. From the earlier study, growing plants in small containers will cause root restriction and reduced photosynthesis rate [8]. Photosynthesis rate could be disrupted by photosynthetic carbon fixation, thylakoid electron transport, stomatal limitation of CO_2_ supply, feedback inhibition by carbohydrate metabolism, and others [9, 10]. The reduction in the photosynthesis rate in root-restricted plants may be mediated by a hypothesis of feedback inhibition mechanism responding to the accumulation of carbohydrate in leaves, which occurs when photosynthetic source capacity is excessive to the sink capacity of the sink organs such as the roots [11, 12]. Carbohydrate accumulation in the leaves tissue may be caused by a reduction in the translocation rate to the available sinks [13].

Besides causing carbohydrate accumulation in the source organs, such as the leaves, restricted root growth could cause a reduction of the sink capacity in the sink organs, such as the roots. On the other hand, the manipulation of source and sink balance was studied by removing sink organs, such as flowers, fruit, or truss [14–16]. However, the effect of root restriction was not identical to the removal of sink organs from the plant materials because root restriction should affect sink organs of roots in particular, since roots are mainly present within the container [17].

Root restriction increased leaf starch content in cotton [18], peach [19], cabbage [20], and loblolly pine [21] probably due to the lack of active sinks [22]. However, a lower photosynthesis rate in small containers was not always related to an increased starch concentration, such as found in the leaves of loblolly pine seedlings [21]. Moreover, the accumulation of carbohydrate in the plant organs depended on the stage of growth, for instance, the accumulation of higher carbohydrate level in the leaves of tomato occurred when the containers were almost filled with roots at 28 days-after-transplanting (DAT) while in the stem at 42 DAT [22].

Root restriction caused an alteration of the sink strength of roots and ultimately, the source and sink relationship of the whole plants. However, in the presence of developing fruits, sink strength could be altered by fruiting while there was an increased in the photosynthesis rate [14] and reduced carbohydrate content of leaves [23]. In a comparison of fruiting and nonfruiting root-restricted and unrestricted cucumber plants, Kharkina et al. [24] had found that the stem had become the most predominant sink in nonfruiting root-restricted plants. They also found that the pattern of dry matter allocation between plant organs was similar between restricted and unrestricted fruiting cucumber plants.

The main cause of growth reduction of plants subjected to root restriction remains unclear since the response cited is the consequence, but not the cause of growth reduction. Several studies have been conducted on the effect of root restriction on the photosynthesis rate and carbohydrate content in different plant parts on vegetable crops, such as tomato [11] and soybean plants [17], as well perennial fruit tree such as peach [19]. This study revealed the potential benefit of soilless substrate saving in a small container for chilli production; however, little information was available concerning the effect of root restriction on physiological changes associated with the growth reduction of chilli grown in soilless culture. Hence, this study was undertaken to determine the effect of root restriction on dry matter partitioning of source and sink organs, leaf gas exchange, carbohydrate content, and yield of chilli plants grown in soilless culture.

## 2. Materials and Methods

### 2.1. Plant Materials, Cultural Conditions and Experiment Site

Seeds of chilli plants (*Capsicum annuum *Kulai var.) were raised in a glasshouse on trays filled with peat moss. Four weeks after germination, seedlings with four true leaves that were uniform in size, were selected and transplanted in polyvinyl-chloride (PVC) columns, which were filled with a mixture of coconut coir dust and empty fruit bunch compost (70 : 30, v : v). The plants were irrigated twice daily by the drip irrigation system, while the volume of the applied nutrient solution varied according to the plant's age (Appendix A, see Table 1). EC of the given nutrient solution ranged from 0.5 to 2.5 dS/m. The nutrient solution formula was based on Cooper's nutrient formulation recommendation (Appendix B, see Tables 2 and 3: [25]). Once a week, the plants were flushed with tap water to prevent salt built-up in the root zone system. The experiment was carried out under rain shelter at the Institute of Tropical Agriculture Protected Complex, Taman Pertanian Universiti, Universiti Putra Malaysia from July to November 2011.

### 2.2. Treatments

Chilli plants were grown in PVC columns of different sizes, which represent different root restriction treatments. Plants that grow in large containers with substrate volume of 9570 cm^3^ were designated as unrestricted control treatment, based on the previous experiment that showed no symptom of root restriction in plants subjected to containers larger than 6831 cm^3^ and smaller than 10557 cm^3^. Meanwhile, root restriction treatment was achieved by growing plants in small containers with a substrate volume of 2392 cm^3^. The treatments with the physical specification of the container are presented in Table 4.

### 2.3. Data Collection.

#### 2.3.1. Growth Measurements

Plant growth and development was monitored by the measurement of plant height and total leaf area. Plant height was measured from the ground level to the shoot tip using a measuring tape. Plant height was obtained through the measurement of four plants from each treatment, started on days 7, 14, 21, 28, 35, 42, 56, 70, 84, 98, and 112. The total leaf area was measured with an automatic leaf area meter (Li-3000, Li-cor Inc., Lincoln, NE, USA). Four plants representing four replications were sampled from each treatment to measure the total leaf area on days 7, 28, 42, 56, 70, 84, 98, and 112.

#### 2.3.2. Dry Matter Production and Partitioning

Plants were partitioned into leaves, stems, and roots to determine the dry weights of individual plant parts. The dry weights of the leaves, stem, and roots were obtained using an electrical weighing balance (TX3202L, Shimadzu Corporation) after oven drying the plant parts at 65°C for 72 hours. The total plant biomass was calculated from the total dry weight of leaves, stem, and root. Four representative plants representing four replications were sampled destructively from each treatment on days 7, 14, 28, 42, 56, 70, 84, 98, and 112. The root-to-shoot ratio was calculated based on dry weights of shoot and root parts according to the equation [26] as stated below:

(1) $$\begin{array}{c} \text{Root}:\text{shoot}\mathrm{ratio}=\frac{\mathrm{Total}\mathrm{root}\mathrm{dry}\mathrm{weight}}{\mathrm{Total}\mathrm{leaf}\mathrm{and}\mathrm{stem}\mathrm{dry}\mathrm{weight}}. \end{array}$$

#### 2.3.3. Physiological Response

1. *Leaf Gas Exchange*. Photosynthetic rate, stomatal conductance, intercellular CO_2_ concentration, and transpiration rate measurements were made once every two-week interval during the experiment period using an infrared gas analyser model Li-6400XT (Li-cor Inc., Lincoln, NE, USA). Measurements of photosynthesis rate, stomatal conductance, intercellular CO_2_, and transpiration rate were taken from young fully expanded and exposed leaves (third or fourth leaf from the tip) of four plants representing four replications from each treatment, after one hour of watering at 1000 to 1100 h. The measurements were taken on the abaxial surface at CO_2_ flow rate of 400 *µ*mol m^−2^ s^−1^ and the saturating photosynthetic photon flux density (PPFD) was 900 mmol m^−2^ s^−1^.
2. *Chlorophyll Fluorescence*. Chlorophyll fluorescence parameters were measured with a Portable Fluorescence Spectrometer Handy PEA (Plant Efficiency Analyzer Meter; Hansatech Instrument, Norfolk, UK) on fully expanded leaves. Chlorophyll fluorescence measurement was initially taken on dark-adapted leaves for 10 min using leaf clips at between 1000 and 1100 h. The following parameters were assessed: Fo, the initial/minimal fluorescence, which is the measure of the stability of the light-harvesting complex; *F*_v_/*F*_m_, representing the maximum quantum yield of PSII, which in turn is highly correlated with the quantum yield of net photosynthesis, where *F*_m_ is the maximal fluorescence value, and *F*_v_ is the variable fluorescence = *F*_m_ − *F*_o_. The measurement of chlorophyll fluorescence was taken once every two-week interval during the experiment period on four plants representing four replications from each treatment.
3. *Chlorophyll Content*. Total chlorophyll, chlorophyll *a,* and chlorophyll *b* in actively growing leaves of the third or fourth fully expanded leaves from the tip, were determined after extraction in 80% (v/v) acetone/water based on the method described [27]. The determination of chlorophyll content was taken once every two-week interval during the experiment period and the leaves were sampled from four plants representing four replications from each treatment.
4. *Relative Chlorophyll Content*. Relative chlorophyll content was determined on the third or fourth fully expanded leaves from the tip between 0900 and 1000 hours using a hand-held chlorophyll meter (SPAD-502; Minolta Corp., Ramsey, N.J.). Measurement was taken from four plants representing four replications from each treatment once every two-week interval during the experiment period.

#### 2.3.4. Sucrose Concentration

Sucrose concentration in leaves, stem, roots, and fruit was determined using the phenol-sulfuric acid method [28]. The phenol-sulfuric acid method is a broad-spectrum method for carbohydrates, measuring both mono- and polysaccharides. Leaves, stem, roots, and fruit samples of chilli were oven-dried at 65°C for 72 hours, ground into a powder, and stored in airtight containers at room temperature until analysis. Total ethanol soluble sugar was extracted from 200 mg of oven dried samples in 100 ml 80% ethanol, and was allowed to stand for 4 h at ambient temperature. Then, the extract was filtered through Whatman 541 filter paper and 1 ml of the extract was diluted with distilled water to the volume of 10 ml. Then, 0.5 ml of each sample was placed in the test tube, and 0.5 ml of 5% phenol was added. 2.5 ml of concentrated sulphuric acid was added rapidly, the stream of acid being directed against the liquid surface rather than against the side of the test tube to obtain good mixing. The tube was shaken before being placed in a water bath at 30°C for 20 min. The tube was shaken again after removal from the water bath and allowed to stand for 30 min at ambient temperature. The absorbance of the sample was read at 490 nm using a spectrophotometer (UV-3101PC UV-VIS-NIR, Shimadzu, Japan). The soluble sugar in the sample was expressed as mg sucrose g^−1^ dry sample. Three replicates per treatment in different plant organs, including leaves, stem, roots, and fruits were measured for sucrose determination.

#### 2.3.5. Yield and Fruit Characteristics

Fruits were harvested from four plants representing four replications from each treatment at the fruit ripening stage, which started when the fruit changes colour from green to red. Fruits were collected when the first fruit started to change from green into red until 120 DAT. Harvesting was conducted once every three-day interval. A total fruit number was recorded, and total fresh weight of the fruit was weighed using an electronic balance immediately after harvest. The harvest index was calculated as a ratio between fruit biomass and total plant biomass [29] from four plants representing four replications from each treatment.

### 2.4. Experimental Design and Statistical Analysis

The experiment was conducted in Randomized Complete Block Design (RCBD) with four replications. The effects of the treatment were identified using Statistical Analysis System [30]. Two-samples *t*-test was used to compare significant differences between treatments at *P* ≤ 0.05.

## 3. Result

### 3.1. Plant Vegetative Growth

#### 3.1.1. Plant Height

**Figure 1:**
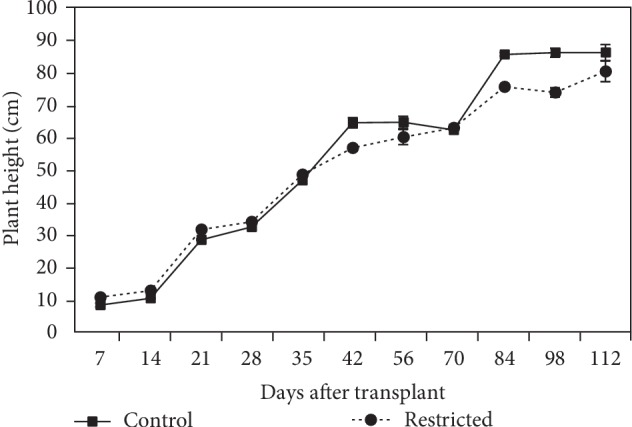
Plant height of chilli plants grown in control (9570 cm^3^) and root-restricted (2392 cm^3^) containers for 112 days after transplanting. Each point represents the mean of four replications ± SE.

Plant height increased with time in both small and large containers, where root restriction significantly (*P* ≤ 0.05) affected the plant height of chilli (Figure 1). On day-7 to day-35, plant height was significantly higher in root-restricted plants compared to control plants. The increase in plant height of root-restricted chilli plants was significantly (*P* ≤ 0.05) inhibited, which began on day-42 and was not significantly different as compared to the control plants on day-56 and day-70. By day-84, there was a sharp increase in plant height in both treatments. Plant height of root-restricted plants was 14% significantly shorter compared to that of the control plants (73.8 vs. 86 cm) on day-98. However, there was no significant difference in plant height of root-restricted plants than that of the control plants on day-112.

#### 3.1.2. Total Leaf Area

**Figure 2:**
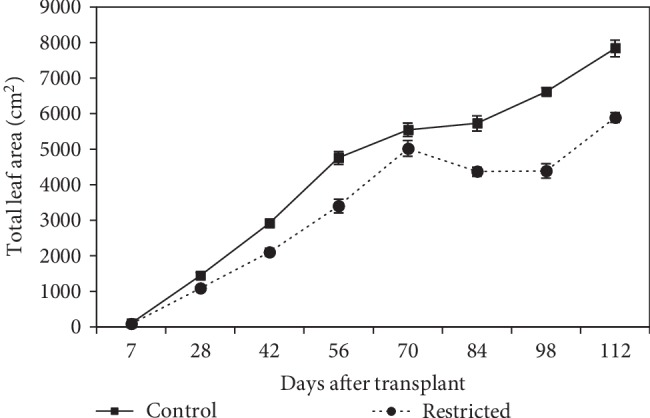
Total leaf area of chilli plants grown in control (9570 cm^3^) and root-restricted (2392 cm^3^) containers for 112 days after transplanting. Each point represents the mean of four replications ± SE.

The total leaf area in the control and root-restricted plants increased slightly after day-7 to day-28 (Figure 2). Total leaf area was significantly (*P* ≤ 0.05) reduced in root restriction on days 28, 42, and 56. On day-70, the total leaf area, however, was not significantly affected by the treatments. From day-84 to day-112, there was a significant reduction of the total leaf area in root-restricted plants. By day-98, there was a sharp increase in the total leaf area in both treatments. Leaf area was significantly reduced by 25% in root-restricted plants compared to that of the control plants on day-112 (5891.83 vs 7837.02 cm^2^).

### 3.2. Dry Matter Production and Partitioning

#### 3.2.1. Dry Matter Production

**Figure 3:**
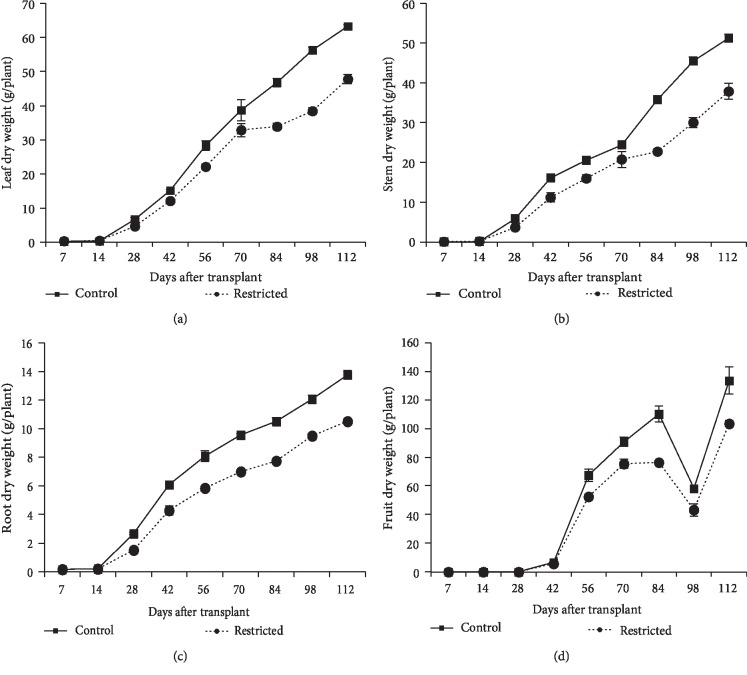
Leaf (a), stem (b), root (c) and fruit (d) dry weight of chilli plants grown in control (9570 cm^3^) and root-restricted (2392 cm^3^) containers for 112 days after transplanting. Each point represents the mean of four replications ± SE.

Root restriction treatment significantly (*P* ≤ 0.05) reduced leaf dry weight and this effect was manifest after day-14 (Figure 3(a)). Root restriction treatment had no appreciable effect on stem dry weight on day-14. By day-28, stem dry weight was significantly (*P* ≤ 0.05) reduced in root-restricted plants until the end of the experiment period (Figure 3(b)). Root restriction significantly reduced the root dry weight, which started after day-14 and this effect was in the same trend with the leaf dry weight (Figure 3(c)). Root restriction significantly affected (*P* ≤ 0.05) fruit dry weight after day-56 of the experiment period (Figure 3(d)). Leaf, stem, root, and fruit dry weight in root-restricted plants were reduced by 24%, 26%, 24%, and 23%, respectively, as compared to those of the control plants by day-112.

#### 3.2.2. Root : Shoot Ratio

**Figure 4:**
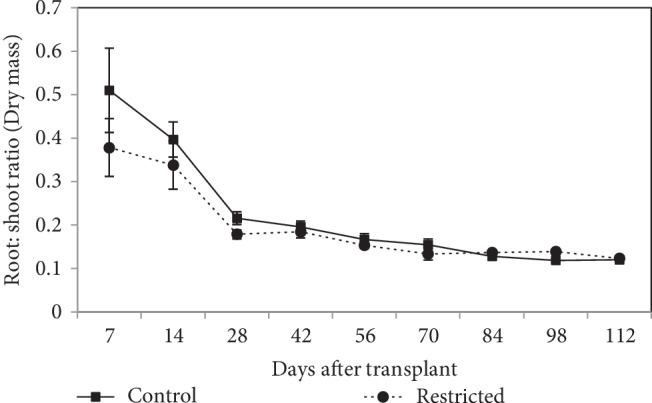
Root: shoot ratio of chilli plants grown in control (9570 cm^3^) and root-restricted (2392 cm^3^) containers for 112 days after transplanting. Each point represents the mean of four replications ± SE.

Root-to-shoot ratio was steadily decreased by time, in restricted and control treatments (Figure 4). Root-to-shoot ratio was not significantly different (*P* > 0.05) in both treatments during the experimental duration, except on day-84 and day-98. At 84 DAT and 98 DAT, the root-to-shoot ratio of root-restricted plants, was significantly (*P* ≤ 0.05) greater than that of the control plant.

#### 3.2.3. Dry Matter Partitioning

**Figure 5:**
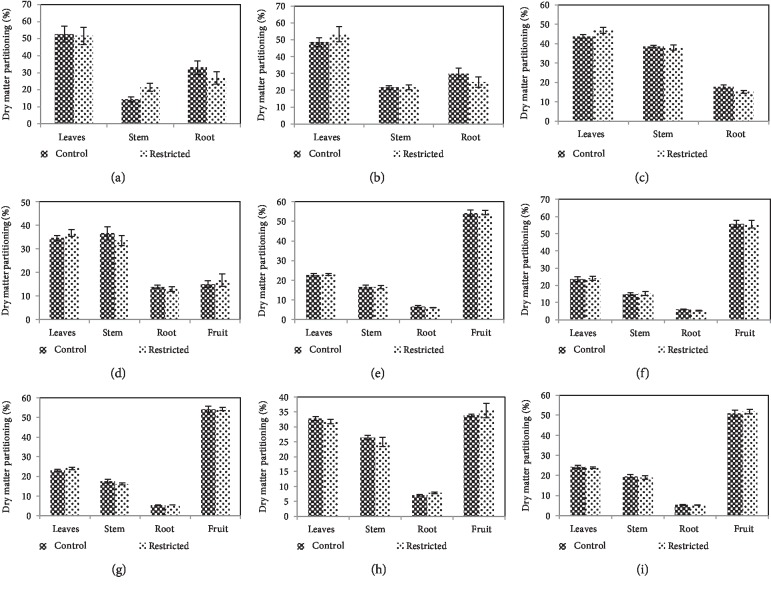
Dry matter partitioning (% of the total dry mass) among organs of chilli plants as influenced by root restriction at 7 (a), 14 (b), 28 (c), 42 (d), 56 (e), 70 (f), 84 (g), 98 (h), and 112 days (i) after transplanting (*n* = 4).

For root restriction and control treatments, the amount of dry matter partitioned amongst leaves, stem, root, and fruit during the experiment period is presented in Figure 5. No significant (*P* > 0.05) difference was found in the assimilation of dry matter in the leaves of root-restricted plants during the experiment period. There was significantly higher (*P* ≤ 0.05) dry matter partitioning in the stem of root-restricted plants only at 7 DAT. However, after day-14 to day-112, dry matter partitioning to the stem was not affected (*P* > 0.05) by root restriction. Dry matter partitioning to the root and fruit was not affected (*P* > 0.05) by root restriction at 112 DAT.

#### 3.2.4. Relative Distribution of Dry Matter

**Figure 6:**
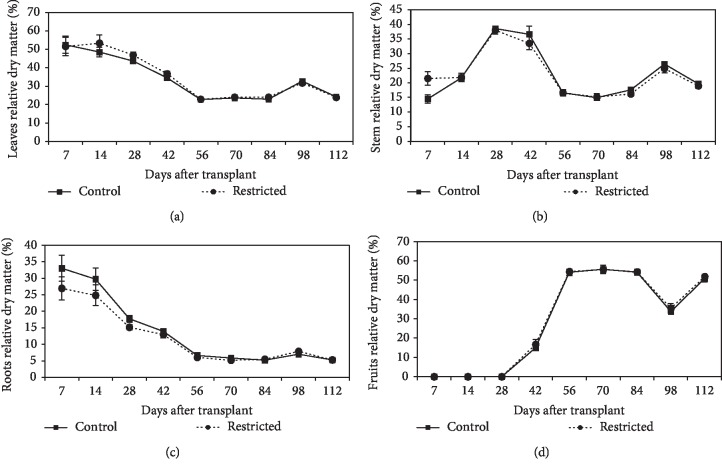
Distribution of dry matter between chilli plant parts as a percentage of the total dry weight for leaves (a), stem (b), roots (c), and fruits (d) between root-restricted and control plants. Each point represents the mean of four replications ± SE.

The relative dry matter distribution of chilli plants between leaves (a), stems (b), roots (c), and fruits (d) is presented in Figure 6. There was a comparatively constant relationship between plant parts in root-restricted plants until day-112. In root-restricted plants, from day-14, there was a steady decline in the leaf and roots parts, whereas the stem showed an enhanced dry weight (Figures 6(a)–6(c)). After the first flowering started to occur within day-15 to day-21, a large amount of growth was accounted for the development of the fruits. Fruit ripening stage occurs within day-64 to day-70, and the matured fruits were plucked. After day-84, there was an increment of the leaves, stem, and roots dry weight because at this time, the chilli plants started to produce new leaves and re-flowered again after the fruits had been harvested from the plants. After day-98, there was a large amount of dry matter produced in the fruits, but a reduced dry matter production in the leaves, stem, and roots. The dry matter production in the roots was less compared to the dry matter production in the leaves and stem. In root-restricted plants, partitioning of dry matter was characterized by comparable dry matter in the leaves, stem, roots, and fruits when compared to that of the control plants. This showed that root restriction did not disrupt dry matter production, as compared to that of the control plants.

### 3.3. Physiological Response

#### 3.3.1. Leaf Gas Exchange

**Figure 7:**
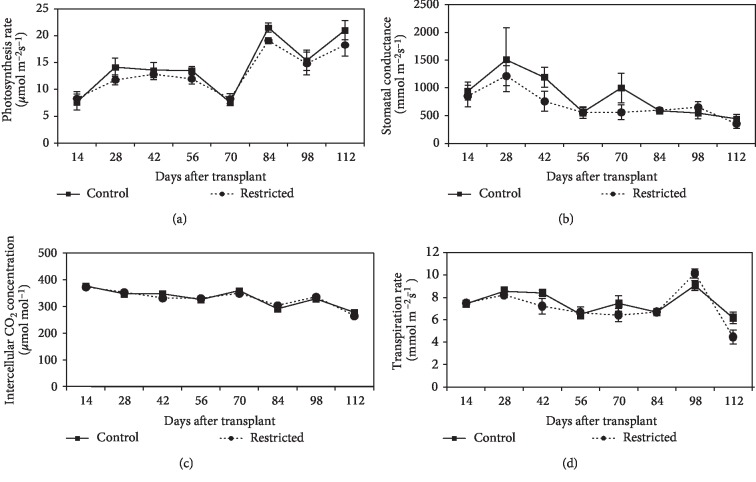
Changes in photosynthesis rate (a), stomatal conductance (b), intercellular CO_2_ concentration (c), and transpiration rate (d) for control and root-restricted chilli plants. Each point represents the mean of four replications ± SE.

In both treatments, the photosynthesis rate was increased after day-14, when the flowering stage started. After day-56, there was a slight reduction in the photosynthesis rate because at this time, the plants started to enter the fruit ripening stage. After day-70, the photosynthesis rate was increased when the plants started re-flowering again. The photosynthesis rate was not significantly (*P* > 0.05) different in both treatments, except on day-84 where the photosynthesis rate was significantly (*P* ≤ 0.05) reduced in root-restricted plants by 11% (Figure 7(a)). Stomatal conductance, intercellular CO_2_ concentration, and transpiration rate were not significantly different (*P* > 0.05) between root-restricted and control plants during the experiment period, as presented in Figures 7(b)–7(d), Similar stomatal conductance between root-restricted and control plants showed that plants may have a mechanism to facilitate the acclimatization in response to the limiting factor.

#### 3.3.2. Chlorophyll Fluorescence

Photochemical parameters, measured through chlorophyll fluorescence, were assessed to test whether photosynthetic acclimation was a result of a reduced irradiance capture at the PSII level. In this study, the maximum PSII photochemical efficiency (Fv/Fm) was not significantly affected by root restriction during the experiment period, except at 84 DAT, and the value of Fv/Fm was below 0.80 regardless of the treatment, as shown in Table 5. This indicated that chilli plants had high stability of the potential PSII photochemical efficiency during root restriction stress. Compared to the control, the initial fluorescence (Fo) in root-restricted plants, was similar (*P* > 0.05) during all the measurement dates, except at 28 DAT, where Fo was significantly (*P* ≤ 0.05) higher in root-restricted plants. The maximal fluorescence (Fm) value in root-restricted plants during all the measurement dates was not significantly (*P* > 0.05) different compared with control plants, except at 112 DAT, where Fm was significantly (*P* ≤ 0.05) reduced in root-restricted plants.

#### 3.3.3. Chlorophyll Content and Relative Chlorophyll Content

In root-restricted chilli plants, chlorophyll *a* and total chlorophyll *a* and *b* were significantly (*P* ≤ 0.05) higher only at 14 DAT, and was similar (*P* > 0.05) with the control plants from 28 to 112 DAT (Table 7). Chlorophyll *b* was significantly (*P* ≤ 0.05) higher in root-restricted plants only from 14 to 28 DAT, and was similar (*P* > 0.05) with the control plants from day-42 onwards. Relative chlorophyll content was significantly (*P* ≤ 0.05) higher in root-restricted plants from 14 to 56 DAT, and was similar (*P* > 0.05) with the control plants from 84 to 112 DAT (Table 6).

### 3.4. Sucrose Concentration in the Leaves, Stem, Root and Fruit

**Figure 8:**
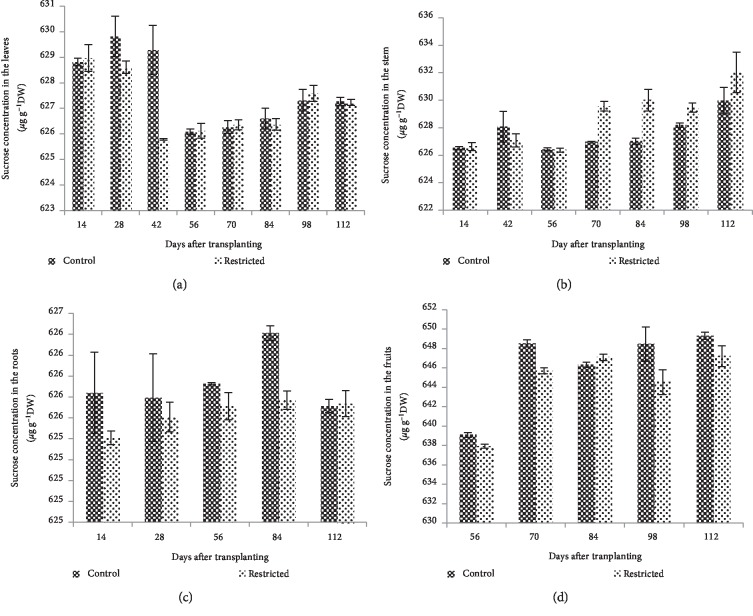
Sucrose contents in the leaves (a), stem (b), roots (c), and fruits (d) of control and root-restricted chilli plants grown for 112 days (*n* = 3).

In root-restricted chilli plants, the leaves sucrose content was not significantly different (*P* > 0.05) compared with the control plants throughout the observation period, except at 42 DAT, where leaves sucrose content was significantly (*P* ≤ 0.05) reduced (Figure 8(a)). Compared to the control, sucrose content in the stem of root-restricted plants was similar (*P* > 0.05) during all the measurement dates, except at 70 to 98 DAT, where sucrose content in the stem was significantly greater (*P* ≤ 0.05) in root-restricted chilli plants (Figure 8(b)). Root restriction treatment had no significant (*P* > 0.05) effect on the sucrose content in the roots of chilli plants, except at 84 DAT, where sucrose was significantly (*P* ≤ 0.05) reduced compared to the control plants (Figure 8(c)). Sucrose content in the fruit of root-restricted chilli plants was significantly (*P* ≤ 0.01) reduced compared to the control plants at 56 and 70 DAT, and was not significantly different (*P* > 0.05) from 84 to 112 DAT (Figure 8(d)).

### 3.5. Yield Production and Fruit Characteristics

#### 3.5.1. Fruit Fresh Weight, Fruit Number and Fruit Dry Weight

Fruit fresh weight, fruit number, and fruit dry weight were significantly (*P* ≤ 0.05) reduced in root-restricted chilli plants compared to the control plants (Table 7). Fruit fresh weight, fruit number, and fruit dry weight in root restriction treatment were reduced by 23%, 17%, and 26% respectively, compared to the control treatment.

#### 3.5.2. Harvest Index

**Figure 9:**
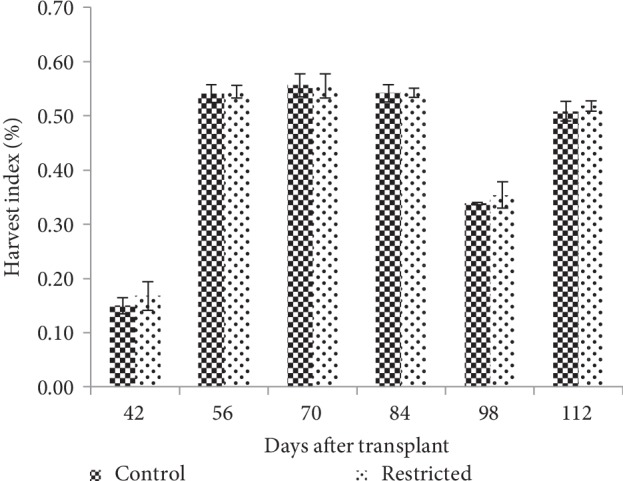
Harvest index of control and root-restricted chilli plants grown for 112 days (*n* = 4).

The harvest index of root-restricted chilli plants was not significantly different compared with the control plants; however, the harvest index was slightly increased in root restriction treatment at 42, 98, and 112 DAT (Figure 9).

### 3.6. Relationship between Growth, Dry Matter Production, Physiological Parameters and Fruit Fresh Weight

There was a strong significant correlation in fruit fresh weight with growth and dry matter production parameters, such as leaf area, leaf dry weight, stem dry weight, and root dry weight under root restriction treatment (Table 8). However, fruit fresh weight had no significant correlation with physiological parameters, such as chlorophyll content, stem sucrose content, and photosynthesis rate. In general, low dry matter production in the leaf, stem, and root would be manifested into low fruit fresh weight as well.

## 4. Discussion

Growth of chilli in terms of plant height and total leaf area was reduced as a result of limited root zone volume throughout the experiment (Figures 1 and 2). The reduction in the total leaf area was attributed to the reduction in leaf and branch number, which reduced the ability of plants to capture photosynthetically active radiation [31]. In the present study, the most important process affected by root restriction is the growth of root cells in a limited space, which caused a 24% reduction of root mass (Figure 3). Accordingly, under ample supply of water and nutrient, leaf area and shoot biomass production were dependant on the size of the root system, based on the strong relationship between leaf area, shoot, and root dry weight (Table 8). Plant height, total leaf area, leaf dry weight, and stem dry weight started to increase at 70 DAT in root restriction because the fruits were plucked when it reached maturity, which shifted the assimilate translocation from the fruits to the production of new leaves and stem. Similarly, Yong et al. [32] found the suppression of leaf area and root dry matter production of cotton, grown under root restriction. A functional balance with shoot growth reduction can be considered as a plant morphological adaptation to cope with root restriction [33].

In this study, plant grown in root restriction had a slightly lower photosynthesis rate as compared to that of the control plants. Reduction in the photosynthesis rate due to root restriction was in agreement with a previous study conducted on tomato by Shi et al. [11]. Reduction of the photosynthesis rate in root restriction implied a reduction of assimilates translocation from the leaves [24]. Reduction of photosynthesis was mainly due to stomatal or non-stomatal factors or both [10, 11, 34, 35]. A stomatal factor was the consequences of depletion of Ci owing to stomatal closure [36]. In this study, root restriction did not affect stomatal conductance, transpiration rate, and intercellular CO_2_ concentration, as compared to the control. The present results did not support stomatal limitation for the reduction of photosynthesis with root restriction, provided there was ample supply of water and nutrient. Non-stomatal factors of the reduction in photosynthesis may be limited by PSII activity [37] or Rubisco activity [34, 38]. The decreased root growth of chilli plants increased the density of relative chlorophyll content from 14 to 56 DAT, probably due to smaller and thicker leaf area. However, chlorophyll *a*, chlorophyll *b,* and the total chlorophyll content were not affected by root restriction. These results contradicted with the findings of Dubik et al. [39], who reported that root restriction reduced nearly half of chlorophyll *a*, chlorophyll *b*, and the total chlorophyll in spreading euonymus, which may vary according to the intensity of the imposed root restriction. However, the total chlorophyll content had a positive relationship with the stem sucrose content. The increased chlorophyll content with higher sucrose content in the stem, showed that sucrose was an important element in stimulating the biosynthesis of chlorophyll production in the leaves [40]. Over 112 days, root restriction did not affect the values of *F*_v_/*F*_m_. Similarly, little effect of root restriction on the maximum photochemical efficiency of PSII was found in tomato [11]. These results suggested that root restriction did not disrupt the stability of the photochemical efficiency of PSII, and also showed no damage to the photosynthetic apparatus within the leaves [41].

The primary function of the roots is for water and nutrient absorption [42]. In root restriction, the involvement of hydraulic signalling depended on the plant species and methods of root zone restriction, which cannot be fully accounted for the shoot growth reduction [6]. The other mechanism involved in suppressed shoot growth of root restriction included reduced cytokinin metabolism between root and shoot [32] and changes of assimilate translocation between root and shoot. The latter mechanism may be related to this study due to the reduction of root sink strength. Photoassimilates are important for growth and energy storage for biochemical activities, and the partitioning process had significant impacts on plant productivity [43].

In the vegetative stage, there was no alteration of dry matter allocation based on a dry weight basis in root restriction, in which around 75% of plant biomass was partitioned to the shoot, with 25% allocated to the vegetative sink (root) (Figure 5). In the generative stage, there was a shift of dry matter allocation, due to the presence of active reproductive sink (fruit), in which around 54% of plant biomass was partitioned to the fruits, with only about 5-6% allocated to the root. In this study, dry matter partitioning between root and shoot was unaffected due to root restriction. Besides, root-to-shoot ratio in root restriction tended to be similar to that of the control, which was similar to NeSmith et al. [44] work on bell pepper. This study indicated that root restriction can maintain an impartial proportion of root growth through direct regulation of shoot growth, probably via the production of hormones in actively growing root apices [45].

In this study, root restriction caused a reduction of sucrose content in the leaves, probably due to the reduction of the photosynthesis rate. Low photosynthesis rate was associated with reduced carbohydrate accumulation and assimilate export in the leaves of root restriction [46]. Previous studies found that the low photosynthesis rate in root restriction was caused by the feedback inhibition of carbohydrate accumulation in the leaves [7, 47, 48]. In this study, no carbohydrate built-up was found in the leaves of root-restricted plants. Similarly, other researchers found that decreased photosynthesis rate due to carbohydrate-induced feedback inhibition did not occur, because carbohydrate concentration was lower in root-restricted plants [11]. In this study, new shoot growth emerged after the harvesting of matured fruits suggesting that continued production of adequate photosynthate and leaves carbohydrate concentration never reached inhibitory levels [49].

In this study, root restriction influenced carbohydrate mobilization, which contributes to the adjustments of photosynthetic activity and translocation of photoassimilates. There were higher sucrose contents in the stem of the root-restricted plants at 70–98 DAT (Figure 8(b)). This result corroborated with a previous study, where a high accumulation of carbohydrate was observed in the stem of tomato [22]. This suggested that after a long period of limited plant growth in root restriction, carbohydrate produced from the photosynthesis process was translocated into the stem. Therefore, vegetative sinks are capable of replacing reproductive sinks in their role for assimilate demand [50]. Plants grown in unrestricted root growth had higher carbohydrate production in the root and fruits than the plants under root restriction (Figures 8(c) and 8(d)). A significant reduction of sucrose content in the roots was found after a long period of root restriction at 84 DAT, which coincided with the significantly reduced photosynthesis rate and photochemical activity of PSII. This was probably because of the non-immediate response of the photosynthesis and photochemical activity of PSII to the shift of source and sink relationship by root restriction. Reduction of root and fruit sucrose contents was probably due to the lower sink strength [46] and sink demand of the roots imposed by root restriction. Besides that, the reduction of carbohydrate production in the roots could be associated with the reduced energy production and energy investment for growth and maintenance of root in root restriction [51].

The productivity of crops is determined to some extent by the allocation of photoassimilates among organs [50]. Fruits were the main sink for assimilates during the reproductive stage [13]. In this study, root restriction had a great influence on the final yield, based on a reduction of about 23% in fruit fresh weight, and 17% in fruit numbers. Reduction in total fruit fresh weight of root-restricted chilli plants was more related to the reduction in the number of fruits and low plant dry matter production, due to lower leaf area (Table 8). Nevertheless, the harvest index in chilli was slightly improved with root restriction, probably due to the presence of active reproductive sink organ, which improved the efficiency of assimilate translocation into the fruit [52]. Similarly, a study conducted by Mandre et al. [19] showed yield efficiency with root restriction on peach, because carbon was preferentially taken from current shoot growth to support fruiting. From this present study's observation, the fruiting efficiency of chilli was enhanced under the root-restricted condition. The results suggested that chilli plants grown in root restriction had more efficient fruits production and managed to overcome the impact of root restriction.

## 5. Conclusion

Root restriction affected plant growth, physiological process, and the yield of chilli plants. Root restriction did not affect dry matter partitioning, but reduced photosynthesis rate and increased sucrose accumulation in the stem. The reduction of plant growth in root restriction was not associated with limited carbohydrate production, but was due to low sink demand from the roots. Root restriction reduced 23% of the yield, with a slightly increased harvest index, demonstrating efficient fruits production in the given small plant size of root restriction. Root restriction can save 50% of substrate volume and would be beneficial in reducing production cost. However, to minimize the yield gap in root restriction, further study should be conducted with focus on the manipulation of the limited root system, probably with beneficial microbes.

## Figures and Tables

**Table 1:** Amount and electrical conductivity (EC).

Day	Amount of nutrient solution (ml day-1)	EC of nutrient solution (dS/m)
1-7	300-500	1.2
8-14	400-600	1.3
15-21	700-800	1.4
22-28	800-1200	1.5
29-35	1200-1500	1.6
36-49	1500-1800	1.8
50-70	1800-2000	2.0
71-120	>1800	2.0-2.8

Note: Standard amount of irrigation recommended by the extension agency, Department of Agriculture, Malaysia.

**Table 2:** Nutrient concentrations (mg/L) for Cooper standard solution used in this study.

Nutrient	Concentration (mg/L)
N (Nitrogen)	200
P (Phosphorus)	60
K (Potassium)	300
Ca (Calcium)	170
Mg (Magnesium)	50
Fe (Ferum)	12
Mn (Manganese)	2
B (Boron)	1.5
Zn (Zinc)	0.1
Cu (Cooper)	0.1
Mo (Molybdenum)	0.2

Note: The solution is based on the Cooper Formulation [25].

**Table 3:** 

Fertilizer / salts	Formula	Weight of salt (g) in
		30 liter water
*STOCK A*		
Calcium nitrate	Ca(NO_3_)_2_. 4H_2_O	6668.67
Ferum EDTA	CH_2_N(CH_2_.COO_2_)_2_FE Na	526.67

*STOCK B*		
Potassium dihydrogen phosphate	KH_2_PO_4_	1753.33
Potassium nitrate	KNO_3_	3886.67
Magnesium sulphate	MgSO_4_.7H_2_O	3420
Manganese sulphate	MnSO_4_.H_2_O	40.67
Boric acid	H_3_BO_3_	11.33
Copper sulphate	CuSO_4_.5H_2_O	2.6
Zinc sulphate	ZnSO_4_.7H_2_O	2.93
Ammonium molibdate	(NH_4_)_6_MO_7_O_24_4H_2_O	2.47

**Table 4:** Treatments with the specification of the container used in this experiment.

Treatment	Specification of container		
	Volume (cm^3^)	Diameter (cm)	Depth (cm)
Control	9570	20	30.48
Root restriction	2392	10	30.48

**Table 5:** Maximum photochemical efficiency of PSII (*F*_v_/*F*_m_), initial fluorescence (*F*_o_) and maximal fluorescence (*F*_m_) for control and root-restricted chilli plants grown for 112 days after transplanting.

Parameters	Treatments	Days after transplant			
		14	28	42	56
*F *_v_/*F*_m_	Control	0.776±0.006a	0.786±0.002a	0.808±0.002a	0.788±0.002a
	Restricted	0.763±0.004a	0.802±0.008a	0.802±0.004a	0.792±0.004a
*F *_o_	Control	593.8±11.59a	576.0±2.82b	595.0±3.58a	603.8±4.87a
	Restricted	607.8±13.92a	597.5±3.97a	503.0±56.63a	605.8±6.03a
*F *_m_	Control	2556.5±58.79a	2691.8±36.74a	3101.5±10.99a	2795.8±41.88a
	Restricted	2562.0±93.89a	2982.5±156.6a	2576.0±297.9a	2799.0±32.55a

		70	84	98	112

*F *_v_/*F*_m_	Control	0.767±0.004a	0.787±0.001a	0.784±0.001a	0.667±0.05a
	Restricted	0.771±0.002a	0.777±0.001b	0.785±0.004a	0.689±0.04a
*F *_o_	Control	579.8±11.52a	572.3±1.44a	594.0±7.01a	675.5±60.26a
	Restricted	589.0±2.38a	581.3±6.47a	593.3±5.81a	552.0±89.10a
*F *_m_	Control	2485.8±80.48a	2704.5±17.35a	2768.5±29.79a	1943.0±150.9a
	Restricted	2548.0±40.17a	2644.3±52.37a	2663.3±53.99a	1402.0±116.5b

Means followed by similar letters within a column for each parameter were not significantly different at *P* ≤ 0.05 based on *t*-test analysis (*n* = 4).

**Table 6:** Chlorophyll a, b, total chlorophyll content and relative chlorophyll content for control and root-restricted chilli plants grown for 112 days after transplanting.

Parameters	Treatments	Days after transplant			
		14	28	42	56
Chlorophyll a	Control	1.208±0.02b	1.514±0.09a	1.651±0.14a	1.811±0.08a
(mg g^−1^fw)	Restricted	1.413±0.06a	1.707±0.05a	1.798±0.13a	1.814±0.03a
Chlorophyll b	Control	0.414±0.01b	0.445±0.04b	0.555±0.05a	0.658±0.05a
(mg g^−1^fw)	Restricted	0.478±0.02a	0.556±0.02a	0.598±0.04a	0.610±0.02a
Total chlorophyll content	Control	1.640±0.02b	1.982±0.11a	2.231±0.19a	2.496±0.18a
(mg g^−1^fw)	Restricted	1.913±0.08a	2.289±0.07a	2.424±0.18a	2.452±0.05a
Relative chl. content	Control	36.18±0.19b	45.23±0.61b	56.00±0.61b	51.65±0.50b
(SPAD unit)	Restricted	37.48±0.39a	48.83±0.26a	58.40±0.69a	58.13±0.48a

		70	84	98	112

Chlorophyll a	Control	1.494±0.03a	1.829±0.09a	2.081±0.09a	1.911±0.09a
(mg g^−1^fw)	Restricted	1.501±0.05a	2.105±0.13a	1.923±0.08a	1.991±0.10a
Chlorophyll b	Control	0.466±0.01a	0.603±0.03a	0.710±0.03a	0.647±0.03a
(mg g^−1^fw)	Restricted	0.462±0.02a	0.617±0.07a	0.662±0.03a	0.693±0.03a
Total chlorophyll content	Control	1.982±0.05a	2.460±0.11a	2.822±0.13a	2.587±0.12a
(mg g^−1^fw)	Restricted	1.985±0.07a	2.753±0.25a	2.614±0.11a	2.714±0.13a
Relative chl. content	Control	58.38±0.15a	59.65±0.57a	60.30±0.61a	60.93±0.28a
(SPAD unit)	Restricted	57.23±0.27b	59.78±0.32a	60.98±0.86a	61.60±0.11a

Means followed by similar letters within a column for each parameter were not significantly different at *P* ≤ 0.05 based on *t*-test analysis (*n* = 4).

**Table 7:** The yield of control and root-restricted chilli plants grown for 112 days after transplanting.

Treatments	Fruit fresh weight (g/plant)	Fruit number	Fruit dry weight (g/plant)
Control	1396.3* ± *25.23a	114* ± *3.64a	250.57* ± *10.06a
Restricted	1070.6* ± *10.39b	94* ± *4.19b	186.41* ± *8.93b

Means followed by similar letters within a column for each parameter were not significantly different at *P* ≤ 0.05 based on *t*-test analysis (*n* = 4).

**Table 8:** Pearson's correlation coefficients matrix among fruit fresh weight, leaf area, leaf, stem and root dry weight, total chlorophyll content, stem sucrose content, and photosynthesis rate at 112 DAT.

Variable	FFW	LA	LDW	SDW	RDW	TCC	SSC	Pn
FFW	1.0							
LA	0.94^∗∗^	1.0						
LDW	0.95^∗∗^	0.93^∗∗^	1.0					
SDW	0.88^∗∗^	0.80^∗∗^	0.93^∗∗^	1.0				
RDW	0.94^∗∗^	0.86^∗∗^	0.94^∗∗^	0.83^∗∗^	1.0			
TCC	−0.15	−0.19	−0.40	−0.41	−0.38	1.0		
SSC	−0.48	−0.41	−0.68	−0.66	−0.66	0.86^∗∗^	1.0	
Pn	0.42	0.18	0.27	0.43	0.39	0.06	−0.009	1.0

FFW: Fruit fresh weight, LA: leaf area, LDW: leaf dry weight, SDW: stem dry weight, RDW: root dry weight, TCC: total chlorophyll content, SSC: stem sucrose content, Pn: photosynthesis rate, ^∗^*P* < 0.05; ^∗∗^*P* < 0.01.

## Data Availability

Regarding on data availability, we would like to declare that all data related to the work provided in the manuscript submitted.

## Appendix

### A. The Total Amount of Nutrient Solution

See Table 1.

### B. Nutrient Concentrations

See Tables 2 and 3.
